# Association of *CYP2A6* Gene Deletion with Cigarette Smoking Status in Japanese Adults

**DOI:** 10.2188/jea.13.176

**Published:** 2007-11-30

**Authors:** Masahiko Ando, Nobuyuki Hamajima, Noritaka Ariyoshi, Tetsuya Kamataki, Keitaro Matsuo, Yoshiyuki Ohno

**Affiliations:** 1 Department of Preventive Medicine/Biostatistics and Medical Decision Making, Nagoya University Graduate School of Medicine.; 2 Division of Epidemiology and Prevention, Aichi Cancer Center Research Institute.; 3 Laboratory of Drug Metabolism, Division of Pharmacobiodynamics, Graduate School of Pharmaceutical Sciences, Hokkaido University.

**Keywords:** cytochrome P450 2A6, genetic polymorphism, smoking behavior

## Abstract

BACKGROUND: Genetic variation of *CYP2A6* is shown to alter nicotine metabolism. This study was developed to investigate the genetic influence of the whole deletion-allele of *CYP2A6* on active and passive smoking behavior.

METHODS: Two hundred and forty Japanese adults, who visited Aichi Cancer Center as outpatients, were genotyped for the wild-type (CYP2A6* 1A, CYP2A6* 1B) and the whole deletion-type (CYP2A6* 4C) polymorphism of *CYP2A6*. Information about active and passive smoking status was obtained by a self-administered questionnaire. Genetic influence of *CYP2A6* polymorphism on smoking behavior was evaluated using the Mantel extension test.

RESULTS: The frequency of the deletion allele was 18%. All 8 subjects carrying two deletion alleles had no smoking habit, and the homozygous deletion genotype showed a tendency to correlate with active smoking status after adjustment for sex and age (p=0.054). However, the proportion of never smokers among heterozygous subjects was almost the same as among subjects carrying no deletion allele (54% and 58%, respectively). Furthermore, *CYP2A6* genotypes were correlated neither with the number of cigarettes smoked per day nor with the age at starting smoking (p=0.364 and 0.880, respectively). Among never smokers, *CYP2A6* genotypes were not correlated with exposure to passive smoking at home or in the workplace (p=0.623 and 0.484, respectively).

CONCLUSION: Despite the possible protection against active smoking behavior in subjects homozygous for the deletion allele, the CYP2A6 polymorphism has only a limited impact on public health because no protective effect was found in heterozygous subjects.

Cigarette smoking remains highly prevalent in many countries, and it is the cause of high morbidity and mortality.^1^ Among various constituents of cigarette smoke, nicotine is the primary compound that establishes and maintains tobacco dependence. The principal metabolic pathway of nicotine is a conversion to inactive cotinine by cytochrome P450 (CYP) 2A6.^2^^,^^3^ The gene encoding this enzyme, *CYP2A6*, has several polymorphic variants.^4^^-^^8^ Large inter-individual differences in nicotine metabolism, including a poor metabolism phenotype with deficient cotinine formation, have been found in humans, and previous works demonstrated that the difference in nicotine metabolism is attributable to the genetic polymorphism of *CYP2A6*.^9^^-^^13^

Considerable ethnic differences exist in the frequency of the inactive alleles of *CYP2A6*. The major inactive allele in Caucasians is CYP2A6*2,^14^^,^^15^ although its allelic frequency is reported to be 1 to 3%. The variant allele, however, was not found in previous studies on Japanese population.^10^^,^^13^^,^^16^ On the other hand, a whole deletion-type of the gene, termed CYP2A6*4C, was reported as the major allele in Japanese lacking CYP2A6 activity.^6^ The allelic frequency of the deleted gene is about 15 to 20% in the Japanese population, which is much higher than in Caucasians.^15^ Thus, Japanese should be a suitable population for studying the genetic influence of the *CYP2A6* whole deletion. Indeed, most poor nicotine metabolizers were shown to be homozygous for the deletion in earlier studies on the Japanese population.^10^^,^^13^

A previous study reported that, in some never smokers, strong symptoms of nicotine toxicity were observed after a subcutaneous injection of 0.6 mg nicotine which produced only half of the blood nicotine concentration typically obtained by smoking a cigarette.^17^ Because subjects deficient in *CYP2A6* activity would be susceptible to nicotine toxicity, it is possible that the genetic polymorphism of *CYP2A6* could affect their smoking behavior. To date, several studies have examined the hypothesis that subjects carrying inactive *CYP2A6* alleles are reluctant to become smokers or at least smoke fewer cigarettes.^20^^-^^22^ These studies, however, have yielded conflicting results, due at least in part to the low frequencies of inactive *CYP2A6* alleles that resulted in very few subjects carrying two inactive alleles.

The present study was conducted to investigate whether the CYP2A6 gene deletion was associated with smoking behavior in the Japanese population. We assessed whether subjects carrying the deletion allele have negative attitudes toward cigarette smoking in the homozygous and heterozygous groups, respectively.

## METHODS

All the subjects in this study were Japanese individuals, who were sequentially recruited when they visited the outpatient clinic of Aichi Cancer Center Hospital for the antibiotic eradication program of Helicobacter pylori infection which was conducted from March through December in 1999.^23^ The study population was chosen simply based on their availability when the present study started. Aichi Cancer Center Hospital is a tertiary cancer institute, and some cancer patients have been informed of their diagnosis a considerable time before referral. Because cancer is a serious health problem, we considered that cancer disclosure would often provide strong influence on subsequent smoking behavior. Thus, out of 283 participants in the program, 241 who had no previous history of cancer were judged to be eligible for this study. Although they included 97 (40% out of 241) participants who stated to be under medications for 107 diseases,^24^ none of them were at a life-threatening level. Written informed consent was obtained from all the participants for gene polymorphism tests without specification of names of polymorphisms and for a self-administered lifestyle questionnaire including smoking habits. All DNA except one could be genotyped, and the remaining 240 outpatients were used for the analysis. Genetic tests for the study subjects were approved by the Ethical Committee at Aichi Cancer Center before the genotyping (Ethical Committee Approval Numbers 12-23).

*CYP2A6* genes were analyzed for the wild-type (CYP2A6*1A, CYP2A6*1B) and the deletion-type (CYP2A6*4C) according to a method reported elsewhere.^16^ In brief, a region ranging from exon 8 of the *CYP2A7* gene or *CYP2A6* gene to a 3′-untranslated region in exon 9 of the *CYP2A6* gene was amplified, and then digested by the restriction enzymes, *Acc*II and *Eco81*I.

Active smoking status was categorized into three levels: never smokers, ex-smokers, and current smokers. The never smokers were those who had smoked less than 100 cigarettes. The ex-smokers were defined as those who had quit smoking at least one year before participation in the study, and the current smokers were those who either were currently smoking or had stopped smoking within the previous year. The current smokers and ex-smokers were asked to indicate how many cigarettes they smoked per day, and were asked the age at which they started to smoke. The ex-smokers were also asked about the age at which they quit smoking. The never smokers were asked to tell whether they were exposed to passive smoking at home or in the workplace.

To investigate the relationship between the proportion of never smokers and demographic variables such as sex and age in 10-year groups, the Pearson chi-squared test was applied. The relationship between the proportion of CYP2A6*4C homozygotes and demographic variables was evaluated in the same way. The Mantel extension test for trend was used to evaluate whether the proportion of CYP2A6*4C homozygotes was significantly correlated with the smoking status (never, ex, current) adjusted for sex and age. In addition, the Mantel extension test adjusted for age was applied to examine the relationship between the proportion of CYP2A6*4C homozygotes and the smoking status among males and females, respectively. The Wilcoxon rank sum test was employed to examine whether the genetic polymorphism of *CYP2A6* (CYP2A6*4C heterozygotes versus others) was associated with the number of cigarettes smoked per day or the age they began smoking, because all the homozygotes found in this study were never smokers. Statistical analyses were performed by SAS^®^ release 6.12 software (SAS Institute Inc., Cary, North Carolina, USA). The test for the Hardy-Weinberg equilibrium was made using STATA^®^ version 7 software (STATA Corp., College Station, Texas, USA).

## RESULTS

A total of 240 subjects, consisting of 119 males and 121 females, were entered onto the study (Table 1). The proportion of never smokers was significantly higher among female subjects (87%) than male subjects (29%) in the study population (p<0.001). The proportion of never smokers was positively associated with age in females (p<0.001), while no association was found in males (p=0.667).

**Table 1.** Demographic characteristics of subjects according to active smoking status.

Sex	Smoking status		
	
Parameter	Current smokers	Ex-smokers	Never smokers
Males			
Number of subjects	45	40	34
Age (year)			
Median	59	61	61
Range	39 - 69	42 - 69	41 - 69
Age at starting smoking (year)			
Median	20	20	NE
Range	16 - 26	18 - 58	NE
Number of cigarettes smoked per day			
Median	20	20	NE
Range	10 - 60	5 - 35	NE

Females			
Number of subjects	12	4	105
Age (year)			
Median	48	55	61
Range	40 - 64	50 - 56	40 - 69
Age at starting smoking (year)			
Median	23	25	NE
Range	20 - 44	20 - 27	NE
Number of cigarettes smoked per day			
Median	20	4	NE
Range	1 - 30	3 - 13	NE

NE: not evaluable.

The genotype distribution for CYP2A6*4C gene was in Hardy-Weinberg equilibrium (p=0.977). Eight subjects (3%), 2 males and 6 females, were homozygous for the *CYP2A6* whole deletion (Table 2), with no sex- or age-related differences in the frequency of the null genotype (p=0.240 and 0.729, respectively). All the subjects homozygous for the gene deletion had no smoking habits, and after adjustment for sex and age, the homozygous deletion genotype demonstrated a tendency to correlate with active smoking status (p=0.054). Because of the sex-related substantial difference in smoking prevalence, we also examined the relationship between the proportion of the homozygous subjects and the smoking status separately in males and females. The homozygous deletion genotype remained to have a tendency to correlate with active smoking status in males (p=0.068), although no such relationship was found in females (*P*=0.328). The proportion of never smokers in heterozygous subjects, however, was almost the same as that in subjects carrying no deletion allele (54% and 58%, respectively). In addition, *CYP2A6* genotypes were correlated neither with the number of cigarettes smoked per day nor with the age of starting to smoke (p=0.364 and 0.880, respectively).

**Table 2.** Active smoking status according to *CYP2A6* genotypes.

*CYP2A6* genotypes	Number of subjects			

	Current smokers	Ex-smokers	Never smokers	Total
CYP2A6*1A/*1A	7 (25%)	4 (14%)	17 ( 61%)	28 (100%)
CYP2A6*1A/*1B	21 (25%)	17 (21%)	45 ( 54%)	83 (100%)
CYP2A6*1B/*1B	13 (27%)	6 (12%)	30 ( 61%)	49 (100%)
CYP2A6*1A/*4C	10 (27%)	7 (19%)	20 ( 54%)	37 (100%)
CYP2A6*1B/*4C	6 (17%)	10 (29%)	19 ( 54%)	35 (100%)
CYP2A6*4C/*4C	0 ( 0%)	0 ( 0%)	8 (100%)	8 (100%)

About a half of the 139 never smokers reported being exposed to passive smoking at home (Table 3). There was no association between *CYP2A6* genotypes and exposure to passive smoking at home (p=0.623). Of the 139 never smokers, 93 had jobs, and the exposure to passive smoking in the workplace was not correlated with *CYP2A6* genotypes among them (p=0.484).

**Table 3.** Exposure to environmental tobacco smoke at home according to *CYP2A6* genotypes in never smokers.

*CYP2A6* genotypes	Number of subjects		

	Exposed	Unexposed	Total
CYP2A6*1A/*1A	9 (53%)	8 (47%)	17 (100%)
CYP2A6*1A/*1B	21 (47%)	24 (53%)	45 (100%)
CYP2A6*1B/*1B	16 (53%)	14 (47%)	30 (100%)
CYP2A6*1A/*4C	7 (35%)	13 (65%)	20 (100%)
CYP2A6*1B/*4C	12 (63%)	7 (37%)	19 (100%)
CYP2A6*4C/*4C	4 (50%)	4 (50%)	8 (100%)

## DISCUSSION

The present study showed that a negative attitude toward tobacco smoking might be associated with homozygosity for the deletion allele, whereas no such protective effect was found in heterozygotes for the deletion. Pharmacological studies have reported that the genotype homozygous for *CYP2A6* deletion was closely correlated with poor nicotine metabolism phenotype.^10^^,^^11^^,^^13^ Nakajima et al. showed that homozygotes for the deletion were completely deficient in cotinine formation.^11^^,^^13^ Probit analysis in their study suggested eventually that there was no poor nicotine metabolizer carrying only one inactive allele. Kitagawa et al. also indicated that the urinary cotinine level in homozygous subjects was only 15% compared with subjects carrying at least one active allele.^10^ These findings may suggest the possibility that the potency of nicotine metabolism is an important determinant of an individual’s smoking behavior. When analyzed separately by sex in the present study, however, the possible association was observed only in males, which might imply the presence of more important factors that affect smoking behavior in females. Indeed, females showed much lower smoking prevalence than males especially in elderly subjects, which would be due in part to widespread social disapproval of female smoking.^25^

Previous studies have shown conflicting results regarding smoking behavior in subjects carrying only one functional allele of *CYP2A6*. Pianezza et al. found less cigarette consumption in subjects carrying at least one copy of the CYP2A6*2 or CYP2A6*3 allele.^18^ Their study, however, was then criticized for the use of inaccurate genotyping that resulted in significant over-estimation of the inactive CYP2A6*2 and CYP2A6*3 alleles.^14^ Subsequent studies, except for that conducted by Rao et al.,^19^ failed to reproduce the correlation between CYP2A6 polymorphism and smoking behavior.^20^^-^^22^^,^^26^ These recent studies, which employed more specific genotyping methods, showed much lower frequencies of inactive alleles with no CYP2A6*3 allele, and most subjects with variant alleles were heterozygous for the functional CYP2A6*1 gene. Our findings also indicate the lack of a significant impact of heterozygous *CYP2A6* deletion on smoking behavior. Because the whole deletion of the gene clearly indicates defective *CYP2A6* enzyme activity, we consider that any heterozygous genotype carrying one functional allele may have no clinical impact on smoking behavior. Further studies assessing the genetic effect of *CYP2A6* should focus separately on populations with the homozygous and heterozygous null genotypes.

If the genetic influence of *CYP2A6* on smoking behavior can be expected only in subjects carrying no functional allele, the magnitude of the protective effects should be small in public health terms. In the present study, the frequency of the whole deletion allele was 18%, which is compatible with findings in other studies on the Japanese population.^10^^,^^13^^,^^27^ Although not examined in this study, the allelic frequency of CYP2A6*2 was considered to be almost zero.^10^^,^^13^^,^^16^ In Caucasian populations, previous studies reported a very low frequency of the deletion allele, and the allelic frequency of CYP2A6*2, a major inactive allele in Caucasians, was up to 3%.^14^^,^^19^^-^^22^ Interestingly, Sellers et al. suggested that CYP2A6 inhibitors could be used as a new approach to treat tobacco dependence.^28^ Such pharmacological approaches may provide a novel means to decrease nicotine dependence and prevent cigarette smoking.^29^

*CYP2A6* has also been shown to metabolically activate a number of tobacco specific procarcinogen nitrosoamines, such as *N*-nitrosonornicotine and 4-(methylnitrosamino)-1-(3-pyridyl)-1-butanone. Therefore, the presence of *CYP2A6* null alleles may theoretically reduce the risk of developing cancer.^30^^-^^32^ A recent case-control study demonstrated a possible protective effect against lung cancer in subjects carrying *CYP2A6* deletion alleles.^27^ It is of interest that subjects heterozygote for the deletion allele had a lower risk of lung cancer than those carrying two CYP2A6*1A alleles in that study. This finding may imply that at least a part of the protective effect is unrelated to the reduction of cigarette consumption because the present study found no difference in smoking behavior between the two populations. Subsequent studies, however, failed to confirm the protective effect of the inactive *CYP2A6* alleles.^22^^,^^26^ This discrepancy may be partly explained by the different frequencies of inactive alleles and insufficient control for confounders, but further work will be needed to address the possible relationship of *CYP2A6* polymorphism with susceptibility to lung cancer.

As an approach to evaluate gene-environment interaction in cancer etiology, a case-only design has often been used to investigate the association between exposure and genotype.^33^ Although this non-traditional approach requires as a prerequisite an independent relationship between exposure and genotype,^34^ the assumption of independence is not always evaluated in advance. We did investigate the relationship between the exposure to passive smoking and *CYP2A6* genotype among never smokers, because never smokers with poor CYP2A6 activity might unconsciously avoid excessive exposure to nicotine and therefore the assumption of independence is not necessarily reasonable. The present study, however, found no association between the exposure to passive smoking and *CYP2A6* genotype, which explicitly showed that a case-only design is applicable in future studies investigating the interaction between them.

The present study is limited in that the study population was not selected randomly from the general population. It should be noted, however, that the overall allele frequency observed in the present study was very similar to the 19.4% reported in the control population, which was accrued as a representative Japanese population, in a large case-control study for lung cancer and *CYP2A6* polymorphism.^27^ In terms of smoking prevalence, the proportion of current male smokers in the present study was lower than that shown in the general population, whereas the proportion of never smokers among male and female subjects did not substantially differ from the figures in the national survey.^35^ Although these characteristics of the study subjects may suggest that the findings in the present study are potentially applicable to other populations, our preliminary results should be confirmed by larger studies, especially those which focus on social, behavioral and personal factors that may affect the acquisition of smoking behavior.

In conclusion, the present study found a possible clinical effect to prevent active cigarette smoking in subjects homozygous for the *CYP2A6* deletion allele. This influence, however, is of limited importance because no protective effect was found in the heterozygous subjects. Since the *CYP2A6* null genotype is closely associated with a poor nicotine metabolism phenotype, pharmacological approaches using CYP2A6 inhibitors seem worthy of further investigation.
